# QUADRatlas: the RNA G-quadruplex and RG4-binding proteins database

**DOI:** 10.1093/nar/gkac782

**Published:** 2022-09-16

**Authors:** Sébastien Bourdon, Pauline Herviou, Leïla Dumas, Eliana Destefanis, Andrea Zen, Anne Cammas, Stefania Millevoi, Erik Dassi

**Affiliations:** Laboratory of RNA Regulatory Networks, Department of Cellular, Computational and Integrative Biology (CIBIO), University of Trento, 38123 Trento, Italy; Cancer Research Centre of Toulouse, INSERM UMR 1037, 31037 Toulouse, France; Université Toulouse III – Paul Sabatier, 31330 Toulouse, France; Cancer Research Centre of Toulouse, INSERM UMR 1037, 31037 Toulouse, France; Université Toulouse III – Paul Sabatier, 31330 Toulouse, France; Cancer Research Centre of Toulouse, INSERM UMR 1037, 31037 Toulouse, France; Université Toulouse III – Paul Sabatier, 31330 Toulouse, France; Laboratory of RNA Regulatory Networks, Department of Cellular, Computational and Integrative Biology (CIBIO), University of Trento, 38123 Trento, Italy; Laboratory of Translational Genomics, Department of Cellular, Computational and Integrative Biology (CIBIO), University of Trento, 38123 Trento, Italy; Laboratory of RNA Regulatory Networks, Department of Cellular, Computational and Integrative Biology (CIBIO), University of Trento, 38123 Trento, Italy; Cancer Research Centre of Toulouse, INSERM UMR 1037, 31037 Toulouse, France; Université Toulouse III – Paul Sabatier, 31330 Toulouse, France; Cancer Research Centre of Toulouse, INSERM UMR 1037, 31037 Toulouse, France; Université Toulouse III – Paul Sabatier, 31330 Toulouse, France; Laboratory of RNA Regulatory Networks, Department of Cellular, Computational and Integrative Biology (CIBIO), University of Trento, 38123 Trento, Italy

## Abstract

RNA G-quadruplexes (RG4s) are non-canonical, disease-associated post-transcriptional regulators of gene expression whose functions are driven by RNA-binding proteins (RBPs). Being able to explore transcriptome-wide RG4 formation and interaction with RBPs is thus paramount to understanding how they are regulated and exploiting them as potential therapeutic targets. Towards this goal, we present QUADRatlas (https://rg4db.cibio.unitn.it), a database of experimentally-derived and computationally predicted RG4s in the human transcriptome, enriched with biological function and disease associations. As RBPs are key to their function, we mined known interactions of RG4s with such proteins, complemented with an extensive RBP binding sites dataset. Users can thus intersect RG4s with their potential regulators and effectors, enabling the formulation of novel hypotheses on RG4 regulation, function and pathogenicity. To support this capability, we provide analysis tools for predicting whether an RBP can bind RG4s, RG4 enrichment in a gene set, and de novo RG4 prediction. Genome-browser and table views allow exploring, filtering, and downloading the data quickly for individual genes and in batch. QUADRatlas is a significant step forward in our ability to understand the biology of RG4s, offering unmatched data content and enabling the integrated analysis of RG4s and their interactions with RBPs.

## INTRODUCTION

RNA G-quadruplexes (RG4s) are non-canonical structures that are increasingly recognized as fundamental post-transcriptional regulators of gene expression (1). RG4s are four-stranded elements composed by stacks of guanine tetrads (called G-quartets) kept together by Hoogsteen hydrogen bonds. The folding of these structures can be controlled by their protein interactors, cations and small molecule ligands, making RG4s highly dynamic. Furthermore, these elements are widespread in the transcriptome and particularly enriched in the untranslated regions of mRNAs and in non-coding RNAs (2,3).

Thanks to their dynamicity and wide array of protein interactors, RG4s can affect cell physiology and pathology. Indeed, these elements regulate all post-transcriptional steps, from splicing to transport, processing, and degradation, acting both in cis and in trans to control protein synthesis (1). Consequently, alterations of the RG4-mediated regulation of the RNA life cycle are associated with several diseases, ranging from cancer to neurodegenerative diseases such as ALS/FTD and Alzheimer's (4). In particular, they have been found to be involved with the onset, progression, and therapy resistance in human cancers by others and us (5,6).

How do RG4s exert their role? The current view is that RG4s are dynamic structures whose folding equilibrium and function are driven by RNA-binding proteins (RBPs) (7,8). Our previous work uncovered a set of such proteins as potential interactors of folded and unfolded RG4s (5), suggesting that their regulatory network is far wider than initially expected and could mediate mechanisms of cooperation and competition between RBPs (9,10). Being able to explore the interactions of RG4s with RBPs and the role of RG4s in post-transcriptional control of gene expression is thus paramount to understanding how these post-transcriptional elements are regulated. In turn, this knowledge would allow us to exploit them as potential therapeutic targets against the diseases they are involved in, including cancer.

While a few resources are available for studying RG4s (11–16), none have the breadth of data and the capability to integrate the different data types required to enable such analyses. In particular, some focus on RG4 ligands (e.g. G4LDB (11) and G4IPDB (12)), some on structures (e.g. ONQUADRO (13) and DSSR-G4DB (14)), others on RG4 sequences (e.g. G4RNA (15) limited to experimentally determined RG4s and GRSB2 (16) limited to predicted RG4s in UTRs only). None of these resources include a comprehensive set of experimentally determined (with both low- and high-throughput RG4 identification techniques) and predicted RG4s. Furthermore, key additional genome-wide data such as the now available wide set of RBP binding sites (e.g. from the >200 eCLIP assays in the ENCODE dataset (17)) or associations to cellular phenotypes and diseases are also generally missing. These limits hamper our possibility of reconstructing the interaction network of RG4s and its cellular context, making the discovery of cellular RG4-mediated mechanisms to be exploited as therapeutic strategies difficult.

To address this need and provide a platform allowing the transcriptome-wide, integrated exploration of RG4s, RBPs, and their interactions, we present here QUADRatlas, the RG4 and RG4-binding proteins (RG4BPs) database. QUADRatlas includes thousands of experimentally determined and predicted RG4 elements in the coding human transcriptome, coupled with a curated catalog of RG4-binding proteins and their association with RG4s. Furthermore, we provide a wide collection of RBP binding sites and involvement in functions in RNA biology, biological processes, and pathologies. This rich dataset is integrated by visualization capabilities including a custom genome browser and analysis functions allowing to predict the interaction of RBPs with RG4s, the enrichment of RG4s in a gene list, and the presence of RG4 elements on custom sequences. All the data is easily downloadable for individual genes of interest and in batch. QUADRatlas is freely available at https://rg4db.cibio.unitn.it.

## MATERIALS AND METHODS

### Data collection

Human genomic and transcriptomic sequences and annotations were obtained from GENCODE, version 36 (assembly GRCh38.p13) (18). Links to external databases are composed on the fly wherever possible to ensure future compatibility with these resources.

Gene-disease associations were retrieved from OMIM (19) and DisGeNET (20). OMIM data was filtered to remove entries with phenotype mapping key = 1 and empty phenotype classification. From DisGeNET, only disease entries were considered, removing diseases falling into classes C21, C22, C24 and C26.

Binding sites for human RNA-binding proteins (RBPs) were obtained from ENCODE (17), selecting replicate-merged, IDR-filtered (threshold = 0.05) peaks from eCLIP assays. The obtained sites were annotated to the gene and transcript containing them with ctk (21).

### Experimentally determined RG4s

Coordinates of experimentally determined RG4 elements were retrieved from two published datasets obtained with the RT-stop profiling and rG4-seq techniques (7,22,23). Regions from the rG4-seq dataset were remapped to the hg38 human genome assembly and the sequences verified to ensure correspondence with the original hg19 version. Single-nucleotide RG4s derived by RT-stop profiling were extended by 30 nts upstream and downstream to obtain the most likely region occupied by the whole element. For regions in which the 30 nts extended outside the exon containing the central RG4 nucleotide the coordinates were extended into the previous/next exon.

The BSgenome.Hsapiens.NCBI.GRCh38 v.1.3.1000 and Biostrings v2.54.0 R packages were used to extract the sequences of the RG4 elements. All RG4s were annotated with their position in the containing transcript (e.g. 5’UTR, CDS, etc.) with ctk (21).

### Predicted RG4s

RG4 elements were independently predicted on human 5’UTR, CDS, and 3’UTR sequences derived from UCSC (GENCODE v36, GRCh38.p13 assembly). We used three tools, namely QGRS mapper (score threshold = 19, (24)), pqsfinder (v2.2.0, score threshold = 47 (25,26)), and G4Hunter (score threshold = 1.2, window size = 25, (27,28)). Parameters were selected by considering the respective tool authors’ indications and assessing the most commonly used thresholds for similar types of sequences, and looking at inflection points in the number of predicted elements, likely to indicate a decrease in the number of false positive predictions. To allow comparing scores between the three tools, scores were normalized in the 0–100% range by considering the minimum and maximum scores that could be output by each algorithm. We also computed the percentile for the score of each predicted RG4 within each tool to evaluate the prediction significance.

Finally, we computed the pairwise intersection of RG4 elements predicted by the three algorithms using R. With these intersections, we thus assembled a ‘prediction consensus’ set of coding transcriptome regions predicted to be an RG4 element by all the tools. The score for a consensus RG4 was eventually computed as the mean of the normalized scores of the three tools for that element.

Transcriptomic coordinates were converted to genomic coordinates with the AnnotationHub v2.18.0 and ensembldb v2.8.1 (29) R packages. The BSgenome.Hsapiens.NCBI.GRCh38 v.1.3.1000 and Biostrings v2.54.0 R packages were used to extract the sequences of the predicted RG4 elements. All RG4s were annotated with their position in the containing transcript (e.g. 5’UTR, CDS, etc.) with ctk (21).

### RG4-binding proteins catalog

RG4-binding proteins (RG4BPs) were determined based on previously published RNA pull-down experiments (5,30–36). These datasets were complemented with curated RG4BPs derived from the literature through two rounds of verification. We extracted all publications from NCBI PubMed matching the search query: [((RNA binding protein)) AND ((G4) OR (g-tract) OR (quadruplex) OR (quartet) OR (guanine rich) OR (g rich) OR (g-quadruplex) OR (g-quartet) OR (g-rich) OR (guanine-rich) OR (poly(G)) OR (GGG) OR (GGGG*))]. The abstracts were checked to select relevant articles, and the full text of these publications was manually examined to ensure that the article provided experimental evidence for RG4–protein interactions. Each RG4BP was then annotated for being previously known as an RBP using RBPbase (https://rbpbase.shiny.embl.de/). Finally, for each RG4BP, a second search was performed in PubMed using the search query: [((Protein Name)) AND ((G4) OR (g-tract) OR (quadruplex) OR (quartet) OR (guanine rich) OR (g rich) OR (g-quadruplex) OR (g-quartet) OR (g-rich) OR (guanine-rich) OR (poly(G)) OR (GGG) OR (GGGG*))]. This second search aimed to identify the RG4s bound by these proteins, the localization (e.g. 5’UTRs, CDS, etc.) of the RG4 in the transcript, and the role of the RG4-protein interaction in RNA biology, biological processes, and diseases. The identified RNA biology functions and biological processes were annotated to Gene Ontology terms with the GO.db v.3.10.0 R package. Finally, each RG4BP was mapped to external databases providing information on the protein structure, modifications, and interactions (Uniprot (37), SMART (38), Phosphosite (39), STRING (40) and Biogrid (41)).

### Database and web interface implementation

QUADRatlas runs on an Ubuntu Linux server 20.04.3 LTS machine. The data is stored and managed by a PostgreSQL v12.11 database, whose schema can be found in Supplementary Figure S1. The back-end runs on the Django v3.2.9 web framework, while the Angular v13.0.3 platform and the Angular Material UI component library v14.0.2 were used to build the graphical user interface. Finally, the genome browsers were implemented with the JBrowse 2 v1.7.10 component (42). The QGRS mapper (24), pqsfinder (25,26), and G4Hunter (27,28) tools were downloaded from the website indicated in the respective publication to be used for RG4 prediction in user-specified sequences.

## RESULTS

### Database content

QUADRatlas has been implemented as a relational PostgreSQL database, coupled to a reactive, app-like web interface. Its current version contains 60616 human genes corresponding to 231957 transcripts. It includes 217 424 RG4s, 42 563 of which (19.6%) are experimentally derived and 174 861 (80.4%) represent the coding transcriptome-wide predictions obtained with three tools and their consensus. We compiled a catalog of 1117 RNA-binding proteins which bind to RG4s (RG4BPs), both in their folded or unfolded conformation. Out of these 1117 RG4BPs, 1089 were identified through 8 previously published RNA pull-down datasets (5,30–36), while 28 were obtained through the manual curation of 95 publications on the subject (see Methods). Whenever known, we associated RG4BPs to the RG4s they bind, obtaining 34 737 such interactions. RG4BPs are annotated for the RG4-dependent RNA biology functions and biological processes they are known to exert, totaling 1175 entries. Finally, RG4BPs and all genes were also connected to the diseases they are involved with (66571 annotations).

Recognizing that the interaction of RG4s with RBPs is key to their function, we then complemented known RG4BP–RG4 interactions with the extensive dataset of ENCODE eCLIP assays (17), including 818 231 binding sites for 148 RBPs in two cell lines (HepG2 and K562).

**Figure 1.**
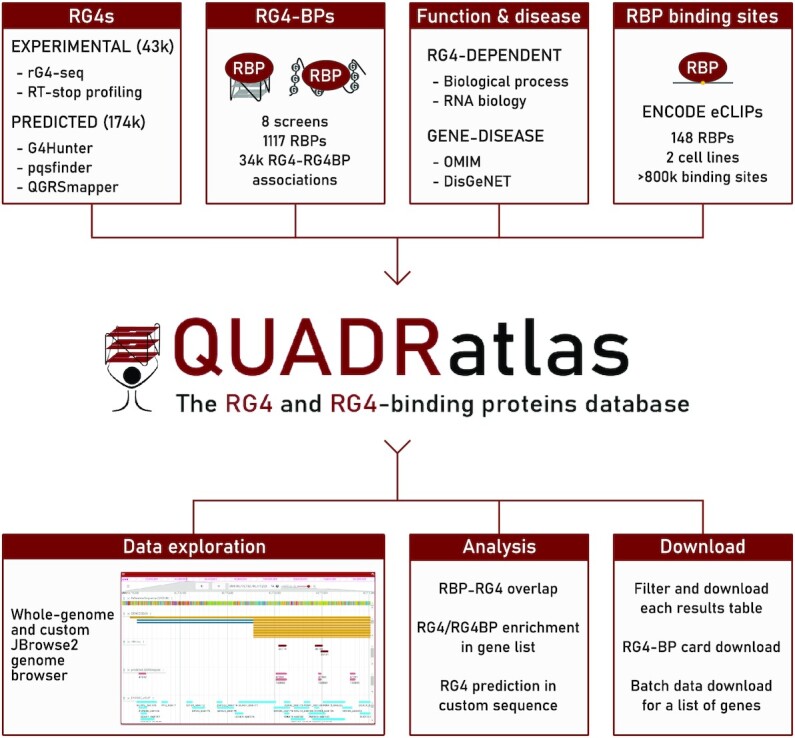
Data and analysis features offered by QUADRatlas. The core of the database consists of experimentally derived and predicted RG4s coupled with an extensive catalog of RG4-binding proteins. These proteins can bind to folded or unfolded RG4s and were obtained by collecting and integrating eight screens for RG4 interactors (see Methods). Functional annotations and eCLIP-derived RBP binding sites are also included to empower hypothesis generation. On top of this rich dataset, QUADRatlas offers several visualizations (e.g. a custom genome browser) and analysis capabilities (e.g. RBP–RG4 overlap analysis), coupled with highly flexible options for downloading the data it contains.

An overview of the database content and features is presented in Figure 1.

### RG4 prediction

**Figure 2.**
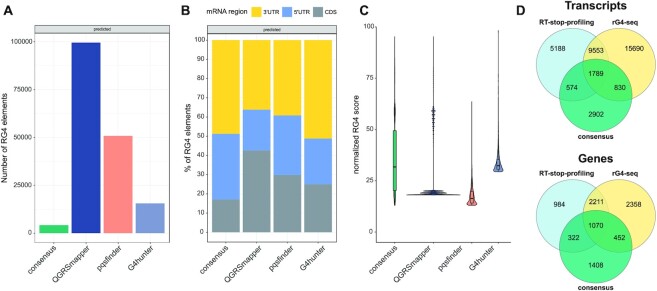
Prediction of RG4s in the coding human transcriptome. The figure shows the properties of RG4 elements predicted in human mRNAs using the QGRSmapper, pqsfinder, and G4hunter algorithms. (**A**) counts of RG4s predicted by each tool and the consensus set. (**B**) mRNA region location of predicted RG4s. (**C**) normalized score distribution of RG4s predicted by each tool and RG4s in the prediction consensus set. (**D**) intersection of transcripts (top) and genes (bottom) containing an RG4 in the prediction consensus set and in the RT-stop profiling and rG4-seq datasets.

To complement the RG4s derived from the RT-stop profiling (7) and rG4-seq techniques (22,23), we performed a coding transcriptome-wide prediction of these elements using QGRS mapper (24), pqsfinder (25,26), and G4Hunter (27,28). By this analysis, we obtained 170532 predicted RG4s across all human mRNAs. In particular, 105 217 elements were returned by QGRSmapper, 53 383 by pqsfinder, and 16 261 by G4Hunter. To provide a more consistent set of predicted RG4s, we computed the positional intersection of elements predicted by the three algorithms, thus obtaining a consensus set of 4329 RG4s (Figure 2A). Most predicted RG4s are located in the UTRs for all three algorithms (57.4–75.05%), with the share of elements in the CDS decreasing further (17% only) when considering the prediction consensus set (Figure 2B). The RG4s in the consensus set are the high-scoring fraction of those predicted by QGRSmapper and pqsfinder, and are comparable to G4Hunter predictions (Figure 2C). While the overall positional reproducibility is limited (the consensus is <3% of all predicted RG4s), high confidence predictions are more consistent. Also, if looking at the gene level rather than at the position (i.e. genes containing at least one predicted RG4), we found that 83% of genes identified by G4Hunter to contain RG4s are common to all three tools, with only 8% of genes that have an experimental RG4 not having a corresponding predicted RG4 (likely reflecting false negative predictions). Finally, we analyzed consistency at the transcript/gene level by intersecting RG4-containing transcripts/genes in the prediction consensus set and the two experimental techniques (RT-stop profiling (7) and rG4-seq (22,23)). This shows (Figure 2D) that half of the predicted consensus RG4-containing transcripts/genes are shared with at least one of the techniques (52.3%/56.7%). Almost one third is also shared with both (29.4%/32.9%).

### Analysis functions

Along with its data exploration features, QUADRatlas offers four analysis functions to support the generation of hypotheses about RG4s and their interactions with RG4BPs.

The RBP-RG4 overlap analysis requires the user to specify an RNA-binding protein of interest and select the source of RG4s to be considered (e.g. rG4-seq). This function then computes the positional overlap of the binding sites of the selected RBP with the RG4s, returning a downloadable table of overlaps and a custom genome browser. This analysis can thus predict whether that RBP can systematically bind to RG4s and identify those specific elements it might interact with.

The RG4 enrichment analysis starts from a list of genes/transcripts of interest to the user (e.g. differentially expressed in an RNA-seq experiment) and a source of RG4s to be considered (e.g. prediction consensus). The function then computes Fisher's exact test for the enrichment of RG4s in those genes with respect to the transcriptome background. Along with the statistics, all the considered RG4s are also output. This analysis can thus predict the involvement of RG4s with the phenotypes that produced the list of genes.

The RG4BP enrichment analysis also starts from a list of genes/transcripts of interest. The user can choose to use known RG4BP-RG4 interactions (thus using only interactions that are known to be mediated by RG4s), eCLIP-derived binding sites for RG4BPs, or both. The function then computes the enrichment of targets for each RG4BP in the gene list via a Fisher's exact test. It thus predicts the regulatory potential of an RG4BP over the phenotypes that produced that list of genes.

Finally, the RG4 prediction analysis recognizes the need to understand whether a specific, user-provided sequence could contain an RG4. To meet this need, this function performs the de novo prediction of RG4s on a user-input sequence with QGRS mapper (24), pqsfinder (25,26) and G4Hunter (27,28). Results are presented in a custom sequence browser and are fully downloadable.

### Data download

QUADRatlas offers several ways to obtain part or all of the data it contains. First, the whole database in SQL format can be downloaded from the website's Download page. On the same page, our users can also obtain an annotated table of RG4-binding proteins, including phenotypes and links to other databases. When using a search or an analysis function, individual results tables are always downloadable as CSV files. Finally, the batch download function allows downloading all data included in the database for a user-specified list of genes. Each dataset (genes annotation, RG4s, RG4BPs association and binding sites) will be output in a tab-separated file consisting of several tables, straightforward to inspect but also machine-readable.

### Web interface and usage

**Figure 3.**
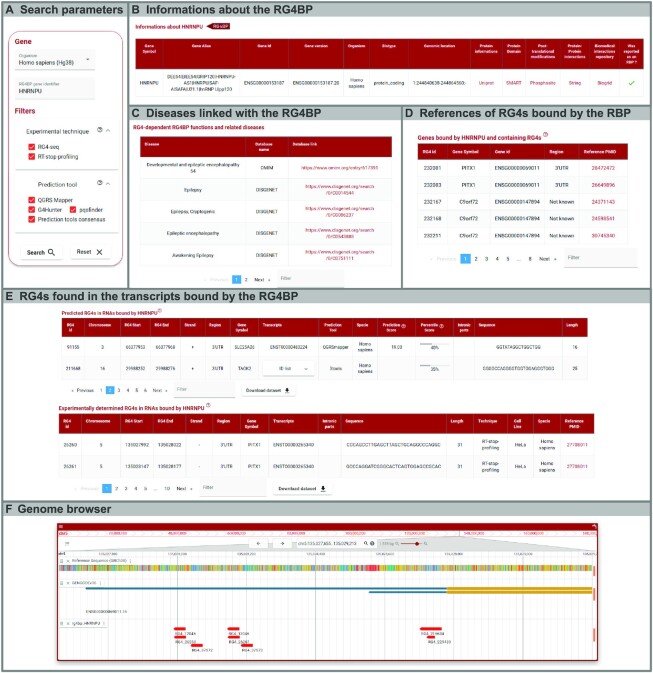
QUADRatlas user interface. The figure shows an example search for an RG4-binding protein. (**A**) Parameters selection box, configured to search for RG4s bound by the query RG4BP within those obtained by high-throughput techniques and predictions. The function will return (**B**) basic information about the query RG4-BP, (**C**) diseases related to it, and, if known, its associated biological functions. Then, (**D**) details about RG4-containing RNAs bound by the RG4-BP are given, and (**E**) RG4s found in these RNAs are retrieved from the experimental and predicted data in QUADRatlas. All tables can be sorted by the column of choice, filtered by keyword using the filter box, and downloaded by clicking on the dedicated button. Finally, (**F**) the obtained RG4s can be explored in a genome browser that also presents all the other data contained in QUADRatlas.

QUADRatlas is available at https://rg4db.cibio.unitn.it. Its interface was implemented by using the Angular framework and the Angular Material UI component library to provide a reactive, app-like user experience. The web interface and the database communicate via a REST API served by a Django backend. Figure 3 presents screenshots of the various components of the search and results page for the RG4BP search function.

Each function is accessible through a top menu on the main page and displays instructions coupled with a parameters box allowing the user to configure the analysis (Figure 3A). Results are presented as a set of collapsible panels. Several columns link to external databases, providing additional details on specific features of the data (e.g. post-translational modification of an RG4BP). A red/blue badge indicates whether the searched protein is an RG4BP or a G-rich binding protein (i.e. preferring unfolded RG4s) according to (5) (Figure 3B). Tables within each panel, showing different aspects of the data (e.g. association with diseases), are organized on multiple pages for ease of visualization. Each table can be dynamically sorted by clicking on the column header. Furthermore, a filter box allows to input keywords to select a subset of the results for display and download with the dedicated button (Figure 3C–E). To provide a quick, intuitive understanding of results, percentage values are shown as progress bars, with the actual value shown below. Additionally, a green/red dot accompanies enrichment *P*-values to indicate statistical significance at the *P* = 0.05 threshold.

Results can also be explored via a custom genome browser (implemented via JBrowse2 (42)), including the genome sequence, gene/transcript annotations, and all the RG4-specific data contained in QUADRatlas. JBrowse2 provides the typical genome browser experience and is therefore easy for any user to navigate. Users can customize display options and select which tracks to display through the menu in the top left corner of the browser. A vector image of the current display can also be downloaded in the SVG format for use in publications and presentations (Figure 3F).

Finally, a detailed user manual, including directions for parameter selection and screenshots of all interface components coupled with descriptions of their content, is available on the website Help page.

## CONCLUSIONS

QUADRatlas (https://rg4db.cibio.unitn.it) is a one-stop resource for the analysis of RNA G-quadruplexes (RG4s) and their interactions with RG4-binding proteins (RG4BPs). The database includes a comprehensive catalog of experimentally-derived and computationally predicted RG4s enriched with biological function and disease associations. A broad set of RBP binding sites coupled with RBP-RG4 overlap, RG4 enrichment, and prediction functions provide a yet unavailable level of integration of RG4 knowledge with post-transcriptional regulation datasets. Ultimately, QUADRatlas will allow generating mechanistic hypotheses about the RG4-mediated control of gene expression and its role in disease. This capability will enable exploiting RG4s to develop new, highly-specific therapeutic strategies.

Future expansions of the database will focus first on adding data for other organisms, especially widely used models (e.g. the mouse). QUADRatlas already supports such development and includes basic annotation for *M. musculus*. However, a demanding curation work will be necessary to gather and process all the required data, both experimentally derived and predicted. Another line of work will focus on including non-single-base resolution RG4 datasets such as those produced by G4RP-seq (8) and BG4-seq (43). This objective will require defining a strategy to locate individual RG4 elements in the larger regions obtained with such techniques. Also, we plan to include single nucleotide polymorphisms and indels affecting RG4s, which would provide an additional tool to explore the potential impact of such elements in disease. Furthermore, we plan to extend the overlap function to allow the search for neighboring binding sites, providing an even more sophisticated tool to identify potential RG4–RBP interactions. Finally, the prediction of RBP binding sites could be added to expand the set of RBPs for which we can analyze such mechanisms.

QUADRatlas is a unique platform for the transcriptome-wide analysis of RG4s. We believe it will be extremely useful to improve our understanding of these key regulatory elements and foster the development of strategies targeting disease-relevant RG4s or RG4–RG4BP interactions.

## DATA AVAILABILITY

QUADRatlas is available at https://rg4db.cibio.unitn.it. It requires no registration or login to access the data and use all of its features. Its content is updated in 12-month cycles for new datasets and major features, and every 6 months for bug fixes and minor upgrades.

## Supplementary Material

gkac782_Supplemental_File
