# Supplementary material for: QUADRatlas: the RNA G-quadruplex and RG4-binding proteins database
Source: Nucleic Acids Res. 2022 Sep 16;51(D1):D240–7. doi: 10.1093/nar/gkac782 (PMC9825518; doi:10.1093/nar/gkac782)
Supplement: gkac782_Supplemental_File [file gkac782_supplemental_file.pdf]

Supplementary Figure 1

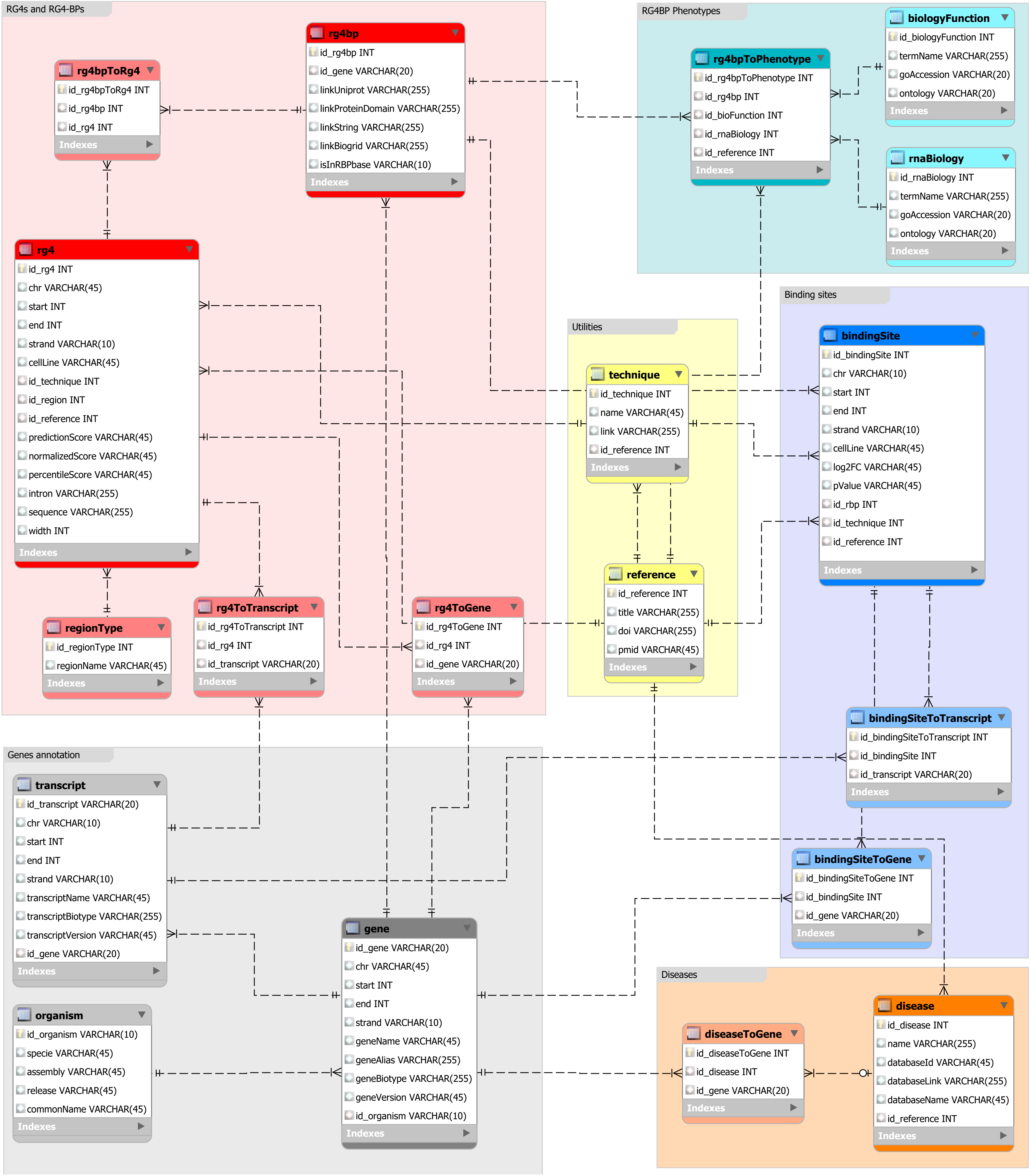

Supplementary figure legends

Supplementary Figure 1: Schema of the QUADRatlas database. *The figure shows the schema of the PostgreSQL database containing QUADRatlas data. The different parts of the model are grouped and color-coded according to the type of data they represent.*
